# Craniospinal cerebrospinal fluid volume changes after extreme bilateral frontotemporoparietal craniectomy and cranioplasty: a volumetric magnetic resonance imaging case report

**DOI:** 10.3325/cmj.2026.67.44

**Published:** 2026-02

**Authors:** Sergej Mihailovič Marasanov, Milan Radoš, Filip Njavro, Miroslav Vukić, Ivana Jurjević, Marijan Klarica

**Affiliations:** 1 Department of Neurosurgery, Unit for Functional and Stereotactic Neurosurgery and Radiosurgery, University Hospital Center Zagreb, Zagreb, Croatia; 2 Croatian Institute for Brain Research, Zagreb, Croatia; 3 Clinical Department of Diagnostic and Interventional Radiology, University Hospital Centre Zagreb, Zagreb, Croatia; 4 Department of Neurosurgery, University Hospital Center Zagreb, Zagreb, Croatia; 5 Department of Neurology, University Hospital Center Zagreb, University of Zagreb School of Medicine, Zagreb, Croatia; 6 Department of Pharmacology, University of Zagreb School of Medicine, Zagreb, Croatia

## Abstract

We report on an 18-year-old woman who sustained severe traumatic brain injury in a traffic accident. The resulting refractory intracranial hypertension required emergency bilateral frontotemporoparietal decompressive craniectomies, which left a large bilateral skull defect. Postoperatively, she developed a bilateral sinking skin flap syndrome, consistent with atmospheric pressure-driven collapse of the cranial compartment. She underwent full neuraxis 3D craniospinal imaging before and three months after elective bilateral cranioplasty using custom implants. Pre-cranioplasty volumetric assessment showed cranial cerebrospinal fluid (CSF) volume of 100.8 mL and spinal CSF volume of 78.2 mL (179.0 mL total). After cranioplasty, cranial CSF volume increased to 152.7 mL and spinal CSF volume to 91.1 mL (243.7 mL total), with an overall CSF net gain of 64.7 mL without relevant change in brain parenchyma volume. Clinically, after neurosurgical and neurointensive rehabilitation, the patient remained neurologically stable, with no new focal deficits, able to continue everyday life, finish high school, and enter university. The volumetric findings suggested global redistribution and expansion of neurofluids within the craniospinal axis when cranial boundaries were restored. Such findings are difficult to explain with the traditional model of CSF production and unidirectional circulation, but align with the Bulat-Klarica-Orešković hypothesis, which views CSF, intravascular and interstitial fluid as a single, hydrostatic, and osmotic capillary-driven system. This case indicates that quantitative craniospinal volumetry after cranioplasty can provide in vivo support for contemporary concepts of CSF physiology and may help guide cranioplasty timing in decompressive craniectomy patients.

Decompressive craniectomy (DC) is a life-saving intervention for refractory intracranial hypertension after severe brain injury. It is usually a last-tier procedure performed after a failure of various less invasive treatment modalities. By transforming the rigid intracranial space into a partially open system, DC drastically changes intracranial and craniospinal pressure-volume relations. After the end of the acute phase and normalization of intracranial pressure, many patients live for weeks to months with a large defect of the calvarial bone. A subset of these patients experience further neurological deterioration, described as the sinking skin flap syndrome or the syndrome of the trephined. This development suggests that the bone defect itself can become an independent pathophysiological factor even in the setting of intracranial normotension.

The classical hypothesis of cerebrospinal fluid (CSF) physiology assumes active CSF secretion from the choroid plexuses into the lateral ventricles, its unidirectional pressure-driven circulation, and absorption via arachnoid villi into dural venous sinuses, all within a fixed cranial compartment. In contrast, the contemporary hypothesis by Bulat, Klarica, and Orešković proposes that CSF and interstitial fluid form a single neurofluid system governed by capillary filtration and reabsorption along the entire craniospinal axis (1-5). From this perspective, large cranial defects represent a rare natural experiment in which cranial compliance and exposure to atmospheric pressure can significantly shift neurofluid distribution between central nervous system capillaries, interstitial fluid (ISF), and CSF.

Experimental work has shown that different body positions can generate a robust hydrostatic CSF pressure gradient across the craniospinal axis in intact animals (6), and recent cranial defect animal models suggest that a bony skull defect can shift intracranial CSF pressure toward atmospheric values in the upright body position while preserving the overall CSF pressure gradient in the craniospinal space. Translating these observations to humans requires in vivo approaches that capture the entire craniospinal system (7). Here, we report on an exceptional bilateral FTP craniectomy in a young adult. Whole neuraxis volumetric magnetic resonance imaging (MRI) analysis was used before and after cranioplasty to quantify the associated changes in cranial and spinal CSF volumes for the first time.

## Case report

**Figure 1:**
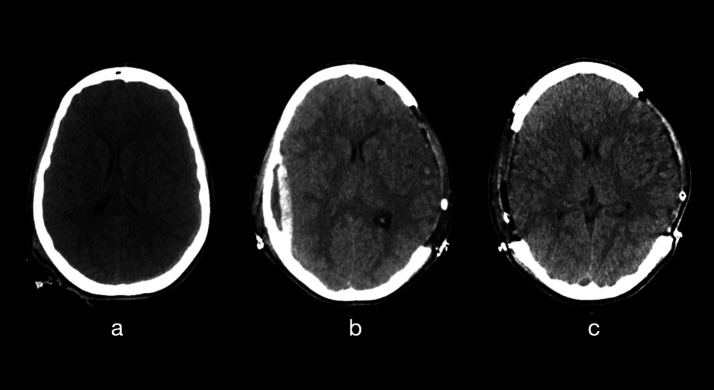
Non-contrast head computed tomography (CT) images of the patient. (**A**) Head CT scan at admission showing a left frontotemporoparietal acute subdural hematoma. (**B**) Postoperative day 1 CT demonstrating a newly formed contralateral right epidural hematoma. (**C**) CT after bilateral frontotemporoparietal decompressive craniectomy showing decompression and extensive bilateral calvarial defects.

An 18-year-old woman sustained a severe traumatic brain injury in a road-traffic accident when she was hit as a pedestrian by a tram. On admission, a non-contrast head computed tomography (CT) scan demonstrated a left frontotemporoparietal (FTP) acute subdural hematoma (aSDH) and extensive skull base and calvarial fractures, without hematocephalus. Whole-body trauma CT revealed bilateral pulmonary contusions and a small right posterior pneumothorax, without abdominal organ injury. Because of the aSDH with signs of refractory intracranial hypertension, she underwent emergency left FTP decompressive craniectomy with subdural hematoma evacuation and placement of an intracranial pressure monitor. Follow-up CT on postoperative day 1 showed a new 20-mm thick right epidural hematoma with mild midline shift. A right FTP craniectomy and evacuation of the hematoma resulted in a very large bilateral skull defect (Figure 1). In the postoperative period, she developed a pronounced bilateral sinking skin flap consistent with atmospheric pressure-driven collapse of the cranial compartment and abnormally increased cranial compliance. The immediate postoperative course was uneventful, and the patient showed an impressive clinical recovery. Early brain MRI showed minor posttraumatic contusions in the left temporal region, without hydrocephalus or new mass lesions. A follow-up 3T brain MRI approximately two months after trauma showed multiple microhemorrhages consistent with diffuse axonal injury (DAI) grade 1. There were no visible territorial infarcts or evident parenchymal losses.

**Figure 2:**
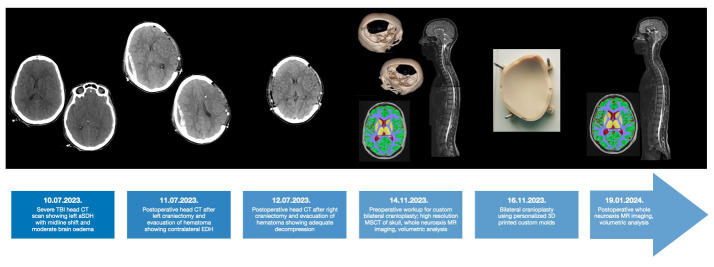
The timeline of key clinical milestones, imaging studies, and cranioplasty workflow from injury through preoperative planning/implant fabrication and postoperative magnetic resonance imaging (MRI) volumetry. TBI – traumatic brain injury; CT – computed tomography; aSDH – acute subdural hematoma; EDH – epidural hematoma: MSCT – multislice computed tomography.

After acute care and rehabilitation, the patient was neurologically stable and scheduled for elective bilateral cranioplasty with custom implants. No CSF diversion procedures were performed. The overall clinical course, imaging timeline, and cranioplasty workflow are summarized in Figure 2.

**Figure 3:**
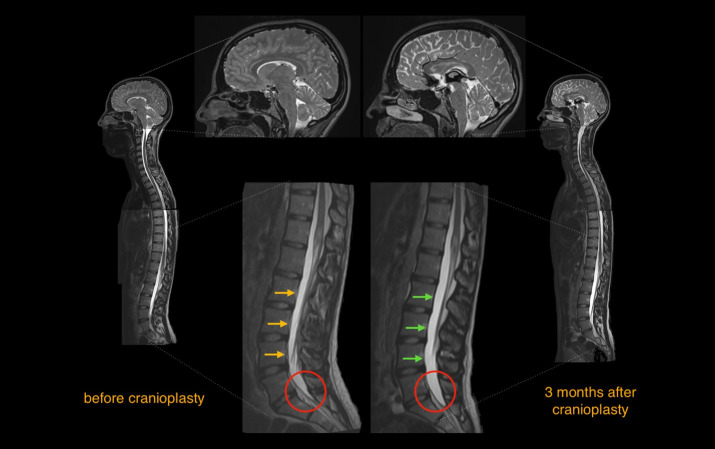
Sagittal whole-neuroaxis magnetic resonance imaging (MRI) obtained before cranioplasty (left) and 3 months after cranioplasty (right) using an identical protocol. Insets, upper row: enlarged sagittal head MRI clearly shows reduced subarachnoid spaces with cranial defects (left) and their restoration upon cranioplasty; lower row: enlarged lumbosacral sagittal MRI – reduced cerebrospinal fluid (CSF) volume provoked the replacement of a retrovertebral area with fatty tissue (yellow arrows) and refilling of the same spaces with CSF after cranioplasty (green arrows). Red circles show marked differences in the fullness of the terminal dural sac in respect to cranial defect vs restoration of cranial integrity.

As a participant in a prospective single-center study of craniospinal CSF volume changes in craniectomy patients performed at a tertiary neurosurgical center, she underwent whole neuroaxis MRI shortly before cranioplasty and three months postoperatively that used an identical imaging protocol (Figure 3). The brain was imaged with 3D T1-weighted magnetization-prepared rapid gradient echo, and cranial volumetric analysis was performed with automated segmentation on the VolBrain platform (8). The volumes of gray matter, white matter, and intracranial CSF were obtained for the pre- and postoperative values. The entire spine was imaged with 3D T2-weighted sampling perfection with application-optimized contrasts, and spinal CSF was segmented semi-automatically using ITK-SNAP v4 (9).

The results showed that brain parenchymal volume remained within the variability range observed in the wider cohort, indicating no relevant change in brain tissue volume between the two volumetric scans. Calvarial bony defects as measured on skull multislice computed tomography had a total area of 138.4 cm^2^ (62.2 cm^2^ + 76.2 cm^2^). Volumetric results are summarized in Table 1.

**Table 1:** Cranial, spinal, and total craniospinal cerebrospinal fluid (CSF) volumes and brain tissue volumes (gray matter, white matter, and total brain) measured before cranioplasty and 3 months later; absolute and relative changes are shown

Parameter	Before cranioplasty (mL)	After cranioplasty (mL)	Absolute change (mL)	Relative change (%)
Cranial CSF	100.8	152.7	+51.9	+51.4
Spinal CSF	78.2	91	+12.9	+16.4
Total craniospinal CSF	179	243.7	+64.7	+36.1
Gray matter (brain)	763	741.2	−21.8	−2.8
Gray matter (brain)	439	435.5	−3.5	−0.8
Total brain	1202	1177	−25	−2.1

Before cranioplasty, cranial CSF volume was 100.8 mL, spinal CSF volume 78.2 mL, and total craniospinal CSF volume 179 mL. Three months after cranioplasty, cranial CSF volume was 152.7 mL, spinal CSF 91 mL, and total CSF 243.7 mL, yielding a net total CSF increase of 64.7 mL (26.5%). The intracranial component increased markedly, by 51.9 mL (51.5%); however, this change was attributable to a net CSF gain, as brain parenchyma volume remained roughly unchanged. In fact, after cranioplasty, brain parenchyma volume decreased by 2%, which indicated a redistribution of interstitial fluid to CSF (and/or minimal tissue loss due to late posttraumatic sequelae). The baseline intracranial CSF volume was lower than expected for a healthy young adult and was consistent with markedly sunken bilateral cranial defects and, consequently, a reduced effective cranial CSF space. Even for this extreme bilateral defect, simple geometric estimates suggested that the volume of the thin convexity CSF layer directly under the 138.4 cm^2^ defect remained clearly below the observed intracranial CSF increase. These estimates were obtained by multiplying the measured defect area by plausible values for convexity CSF layer thickness seen after cranioplasty; even generous assumptions did not approach the measured intracranial gain. As an incidental finding, the spinal MRI showed a completely asymptomatic tumefaction of the medullary conus, which was monitored with sequential MRI imaging. Clinically, the patient remained neurologically stable throughout this period, with no new focal deficits. She was able to continue everyday life, finish high school, and enter university.

## Discussion

In the presented case, the restoration of skull integrity after extreme bilateral FTP craniectomy was associated with a substantial increase in both intracranial and spinal CSF volumes, without a relevant change in overall brain parenchymal volume. Although the patient had minimal chronic focal contusions and DAI grade 1, there was no large territorial infarct or progressive tissue loss that could plausibly account for the magnitude of the observed CSF gains.

Our bedside observation complements previous laboratory experimental animal models of cranial defects (10). In experimental settings, skull defects can modulate the levels of intracranial CSF pressure that are expected in the upright posture toward more positive values, consistent with atmospheric pressure acting through the compliant soft-tissue interface over the defect. Our volumetric findings suggest that the same change in cranial bone integrity may also influence the global craniospinal neurofluid content and its cranial-spinal distribution once skull integrity is restored.

Three aspects of our case are particularly informative. First, a net gain in total craniospinal CSF volume was over 26%, although no CSF was drained or added and no CSF shunt was implanted. In a strict Monro-Kellie framework and according to the classical hypothesis of CSF physiology, one might predominantly expect redistribution between compartments rather than a sizeable net change in total craniospinal CSF volume. Second, the increase in intracranial CSF exceeded any realistic estimate of convexity CSF that could be “regained” directly under the defect. We approximated the potential local contribution by multiplying the measured defect area (138.4 cm^2^) by plausible additional subarachnoid CSF layer thickness under the cranioplasty. Even generous assumptions did not fully account for the measured 51.9 mL intracranial increase. Combined with a simultaneous increase in spinal CSF volume, this suggests a more global shift in neurofluid content following regained intracranial compliance rather than a local effect alone (11). Third, there was a simultaneous parallel net increase in both cranial and spinal CSF volume. This observation is consistent with previous volumetric and hydrodynamic studies that identify the spinal compartment as a major compliance reservoir within the craniospinal system (6,12-16). Our report adds to this literature by showing that by simply restoring the integrity of the rigid cranial box, without any CSF diversion procedure, measurable spinal CSF expansion is induced.

These findings are much easier to interpret within the Bulat-Klarica-Orešković (BKO) concept than within the classic CSF production-circulation model. In the BKO view, intravascular fluid, CSF, and ISF form a neurofluid continuum in which water exchange is governed by Starling forces at capillary walls and is modulated by posture, arterial pulsatility, and mechanical properties of the craniospinal envelope (1-3,5,16). A large skull defect makes the cranial compartment directly exposed to atmospheric pressure and consequently abnormally compliant. This alters the pressure-volume relationship, diminishes transmural arterial pulsatile forces, and results in a pathological shift of neurofluids between its compartments.

Cranioplasty re-establishes the closed cranial vault and normalizes compliance, allowing for physiological transcapillary pressure gradients and arterial pulsatile forces to re-emerge in a physiological fashion. The accompanying spinal CSF gain supports the idea of a coupled craniospinal reservoir, as suggested by shunt studies in idiopathic intracranial hypertension and by posture-related volumetric studies (13-15).

From a clinical standpoint, this case underlines two practical points. First, whole neuraxis volumetric MRI using standardized segmentation tools (VolBrain and ITK-SNAP) offers an objective way to quantify the neurofluid content and to study the consequences of craniectomy and cranioplasty (7). Second, it supports the notion that some neurological or cognitive improvements after cranioplasty may relate not only to mechanical brain protection or isolation of brain parenchyma from atmospheric pressure, but also to restoration of more physiological brain parenchyma perfusion and interstitial fluid exchange with inherent consequences on metabolite exchange (17,18).

The strengths of this case report include the use of standardized whole-axis volumetric MRI at two clearly defined time points and the presence of an unusually large bilateral skull defect in the context of no measurable brain parenchyma loss, where the large exposed area magnifies the neurofluid effects of craniectomy, and later of cranioplasty. The limitations of this report are limited generalizability and the lack of systematic clinical and patient-reported outcome measures. As such, the observations should be viewed as hypothesis-generating.

For clinicians and researchers, the primary take-away lesson from this case is that the magnitude and pattern of CSF volume changes after cranioplasty following extreme bilateral craniectomy are difficult to explain with a purely choroid plexus-driven CSF circulation concept. They are, however, highly compatible with a neurofluid system distributed within communicating compartments (CSF, intravascular, interstitial) whose behavior critically depends on craniospinal integrity and compliance.
